# Medical and Physical Intelligence

**Published:** 1822-10

**Authors:** 


### MEDICAL AND PHYSICAL INTELLIGENCE.

### 1. *Cubebs.*

A Correspondent from Wolverhampton has favoured us with the result of his experience in the treatment of Gonorrhœa with the Java pepper, which is altogether highly in favour of that remedy. As there is nothing novel in the symptoms or progress of the particular case with which he has favoured us, we need only say that the disease was effectually cured in one week; but we feel pleasure in subjoining the following remarks, which we think may contribute still further to extend the use of, what we consider, a very valuable medicine.

From the result of about twenty cases, (the details of which, from the number now published, are uninteresting,) our correspondent deduces the following conclusions:—

That the Piper Cubeba, or Java pepper, is a specific in about nineteen cases in twenty, when administered not exceeding two days after the development of the disease.

The heat in the rectum produced by the pepper is the surest criterion of its dose; for, in most of the patients who took it, the disease rapidly disappeared on the accession of this effect.

The functions of the stomach are not at all injured by a continuance of the cubebs, (as is the case in the administration of the balsam capivi and many other medicines,) but, on the contrary, strengthened; the bowels brought into a regular and healthy action, and the spirits much exhilarated.

The Java pepper is as justly deserving of the name specific for gonorrhœa, when given in the acute stage, as any other medicine which we call specific in its appropriate disease.

The pepper is rendered nearly inert by remaining long in a powdered state, probably owing to the evaporation of its volatile oil.

The best vehicle for the medicine is water, of which about three ounces is requisite when two drachms are to be taken.

The pepper cures gonorrhœa, I conceive, by its increasing the mucous discharge from the intestines, in conjunction with its diuretic properties; for the alvine excretions contained much more mucus during its exhibition, the urine was more abundant, and savoured strongly of the medicine.

2. *Two Cases of Aneurism of the Aorta, not known during Life.* By Mr. A. S. J. BAYLE, House-Pupil at the Maison Royale at Charenton.

The subject of the first case, a man forty-four years of age, who, as a revenue-officer, had suffered much from fatigue, and had also lived a very irregular life,—drank hard and used much mercury,—experienced, for the five years previous to his death, frequent attacks of pulmonary complaints, attended with shortness of breath and a troublesome cough, which within the last twelvemonth became habitual; his voice also acquired a particular hoarseness; and he complained of a deep, but vague, kind of pain in the chest. On the 24th of July, 1820, the following symptoms were present:—the countenance had a bluish tinge; great dyspnœa, always aggravated at night; expectoration of frothy mucus; voice very hoarse, with pain in the larynx; no alteration in the pulse; and a good appetite, but frequent vomitings during meals. During the month of August he rallied a little; but, about the beginning of September, the foregoing symptoms recurred with increased violence, but still there was no change in the circulation or digestion. The chest was at this time examined carefully with the stethoscope, but gave no indication of disease of the lungs or heart: indeed, he never experienced either syncope or palpitation. From a review of all his symptoms, his complaint was pronounced to be *a laryngeal phthisis, with ulceration of the organ of the voice, no lesion of the lungs, and the vomitings proceed from the violence of the cough.* During the whole of this month, the complaint went on progressively increasing. On the 19th of October he died, after three days of great suffering from violent dyspnœa and a sense of suffocation. He continued to the last day to refer to the larynx as the seat of his pain.

On dissection, the right lung was found to be perfectly healthy; the left in the first degree of hepatization, brownish, and hardened throughout its whole extent, and full of a sero-sanguineous fluid, escaping abundantly upon incision; the larynx perfectly sound; the mucous membrane of the bronchiæ slightly red; the pericardium containing six ounces of serum; the heart small and healthy; the aorta dilated at its origin, and presenting in its cavity some irregular bony plates, adhering intimately to its internal coat. The aneurismal tumor was about the size of the fist, oblong, and flattened before and behind, and was situated in the upper and left part of the thorax: its cavity was filled with clots of black blood, and a great number of bony incrustations rendered its parietes very uneven; as was also the circumference of the vessel which opened into the lower part of the tumor. The aneurismal sac adhered to the lateral part of the vertebral column; and on the left side the œsophagus was compressed by it. Besides the coagula, it contained several loose bony scales in its cavity. All the abdominal viscera were sound.

*Second Case.*—Achilles Boutel, fifty-two years of age, had served as a soldier in the colonies a long time, and afterwards returned to his original profession of house-painter, which he was obliged to quit on account of the frequent cholics he experienced. He had been much addicted also to liquor and to venereal excesses; which propensities he retained, and indulged, even in the latter periods of his malady. He had, for fourteen or fifteen years, been subject to the attack of a harsh cough, and was frequently troubled with catarrh during the winter.

The 15th May, 1820, he was attacked with a very sharp pain in the side, with a dry cough, which the application of twenty-five leeches removed; but some pain in the back remained, and which any sudden motion, or the act of stooping, always augmented. On the 13th June, this pain in the back became more violent. He had also a pain on the left side of the chest, always accompanied with a little cough, to which he paid no attention. There was no alteration in the pulse, and the appetite was good. Towards the end of the month, though the symptoms continued, they were milder.

On the 22d of August, the pain became so acute as to affect him at each attempt at expiration; cough recurring by fits, principally during the night; habitual hoarseness of the voice; no fever, or increased heat of skin. Leeches, blisters, a pectoral tisane, and an antispasmodic potion, produced no sensible amendment. The patient still continued to get up every day, but grew weaker. In December, the same symptoms continued; the body was sensibly wasted; but the pulse remained perfectly regular, and not accelerated. In January, 1822, the labour of respiration became very great; the cough had a very peculiar sound. On the 18th a more violent attack ensued; and on the 24th he died.

*Dissection.*—The lungs were found perfectly healthy, excepting at the top, where there were slight adhesions and some tubercles; the bronchiæ contained much mucus, and the membrane was very red. The heart was large, and the left ventricle rather thicker than ordinary. On lifting up the left lung, so as to display the aorta, three considerable tumors were perceived in the course of that vessel: one occupied its arch, about the size of an egg; the middle one, behind the root of the lung, was as large as a fist; and the lower, situated immediately above the diaphragm, was round, and nearly the size of two fists joined together: the two former soft to the touch, the third very hard, externally. The aorta, opened from its origin to below the diaphragm, was full of fluid black blood. The aneurismatic portion presented the following appearances:—the internal coat of the artery was very rough, and felt uneven; it had some bony incrustations also imbedded in the proper coat of the artery. Below the diaphragm the aorta was sound, but its diameter diminished nearly one-third. All the abdominal viscera were sound.—(*Bibl. Medicale.*)

### 3. *Petrifaction in the Corpora Striata, observed by* M. Avisard, D.M.P.

A water-carrier, fifty-seven years of age, a strong and hearty man, suffered for two years severe cramps in the calves of his legs, which recurred from time to time, and were occasionally so painful as to oblige him to keep his bed for a day or two. In the beginning of March, 1818, he had an attack; but this time the cramps were not confined to the legs: the fingers were strongly bent on the hand, the fore-arm on the arm, and the latter brought close to the chest. He had vertigo, and a sense of weight in the head; an inclination to vomit; little sleep; and no appetite. He took four grains of emetic tartar, in two portions, without effect. The next day, a purgative produced *eighty* stools. He was taken to the Hotel Dieu on the 8th of March. His eyes were injected; his face red, and convulsed on the right side; speech and pronunciation extremely difficult; the heat of the body intense; the pulse frequent and full; great thirst; the epigastrium tender to the touch; extending the fore-arm and fingers gave pain, and brought on convulsions; the lower limbs in a painful state of extension; no diminution of sensibility in the limbs. He was bled, and put upon a rigid diet. Fifteen leeches put upon the epigastrium lessened the pain. Next day, the abdomen appeared less full, the face was calm, but no other alteration. Twelve leeches were applied to the neck, and the same number in the evening. The four following days, the symptoms continued much the same: the motions of the hands and arms were, however, more free, and the fever somewhat less. This amendment continued until the fourteenth day, when the fingers of the right hand alone remained bent. On the fifteenth day, deglutition became more difficult; violent fits of coughing came on, convulsions, a sense of suffocation, a burning skin, and a frequent and very small pulse, were the most prominent symptoms. The day following, all these appearances had subsided, and the patient was as on the preceding days. A warm bath and cold affusion upon the head was prescribed, during which convulsions and suffocation came on. In the evening there was no fever, and the fingers of the right hand could be extended. On the following days, the baths and affusions were continued: the same effects always ensued during their administration; but there was no fever in the evening, and the limbs were as pliant as when in health. On the twentieth day, difficulty of respiration came on, violent fever, and a little cough. On the twenty-first, the pulse was scarcely perceptible; rattling in the throat ensued; and that day he died.

*Dissection.*—Head: The arachnoid membrane was white and thickened, especially upon its upper part; the brain very firm; in the corpora striata, about thirty petrifactions were found, from the size of a grain of millet to that of a pea; there was very little serum in the ventricles.—Abdomen: The great curvature of the stomach presented a large brown patch, of the size of the palm of the hand; the mere rubbing with the hand was sufficient to detach the mucous membrane from the others. There was no redness of the intestines, but some of the mesenteric glands were larger than natural.

*Reflections.*—The appearances of the stomach are referred, without hesitation, to the dose of tartar emetic, and to the violent purgative; and to this cause is attributed the difficulty of swallowing and the cough. The petrifactions found in the corpora striata, and which, it is presumed, gave rise to the spasmodic motions of the limbs,—are they not fresh proofs of the difficulty of recognizing, at the bed-side of the sick, the different accidental or organic causes of diseases of the brain? —(*Bibl. Medicale.*)

### 4. *State of the Skin, &c. in Yellow Fever.*

From the facts observed by himself, or recorded by others, Dr. Desmoulins has been led to the following conclusions with regard to the biliary secretion and yellowness of the skin:—

1. That, in yellow fever, there is no increase of the secretion of bile.
2. That the black vomit and dark-coloured stools are the produce of exhalation from the surface of the intestines.
3. That the yellow colour of the skin is produced by the blood in the minute vessels of the cutis, which are in a state of congestion, analogous to that which, in the mucous membranes, gives rise to hemorrhage.
4. That the firmer texture of the skin alone prevents this likewise from ending in a similar discharge.
5. That the yellow colour, which is almost always preceded by petechiæ, is only a sort of general ecchymoses.
6. That the yellow fever is nothing else than a congestion or determination of blood to the skin and mucous membranes simultaneously, principally to those of digestion.

### 5. *Precautions against the Introduction of Yellow Fever.*

In our last Number we ventured to advocate the non-contagious nature of this disease: our opinion has received considerable increase of probability, from a quarter where the practical facts on this subject ought to be well known. At New Orleans, the Society of Medicine have demanded, and procured, the abolition of all the lazarettos, even for the vessels which come from the island of Cuba, where the yellow fever prevails endemically. The following are the grounds of the petition made by the Society:—

1. Yellow fever is not a contagious disease, nor one capable of being transported.
2. The most disastrous maladies have all their principle, their *hotbed,* and residence, in situations where the air is infected by miasmata, exhalations, or putrid evaporations.
3. The only means of opposing these pestilences is to plant the country, and people it with labourers, to draw off the stagnant waters, and to maintain extreme cleanliness.

### 6. *Yellow Fever at the Hotel Dieu.*

M. Bally has communicated to the Royal Academy of Medicine at Paris some explanations on the subject of two cases resembling yellow fever, which recently occurred at this hospital. The first appears to have been a case of hematemesis, of which the patient died after a short illness. This, perhaps, is not to be wondered at, considering the great heat of the season, and the practice in Paris of administering warm wine and other stimulating remedies, which appears to have been done before his admission at that institution. The second laboured under jaundice, but without vomiting; and the examination after death showed extensive disease of the liver and lungs. The occurrence of these two cases in the same ward seems to have given rise to this chimerical alarm, at a time when the public mind in France is morbidly alive to apprehension on this subject. We had lately a report of the plague existing in London, without even so much foundation for its origin.

### 7. *Treatment of the Barcelona Fever.*

Dr. FRANÇAIS has read to the Academy of Medicine a memoir on this subject. Blood-letting, which has been frequently employed and regarded as highly useful in America, has always proved deleterious in Spain. The tonic and revulsive treatment has succeeded best, particularly the exhibition of cinchona, and the application of the moxa along the spinal column. It may readily be supposed that the means of employing these universally did not exist, but, according to Dr. Français, the trials made were sufficient to demonstrate the happy effects resulting from this plan.

### 8. *Application of Auscultation to the Study of Pregnancy.*

We formerly noticed M. KERGARADEC's remarks on this subject: these have since been reviewed by M. DUGES, who asserts that he has tried it on twelve women without success. To this M. K. has written a reply, of which the following is an extract:—"To his negative I oppose the positive testimony of Messrs. the Professors Recamier, Béclard, Désonneaux, the Doctors Laennec, Delens, Deneux, Ducamp, Foderà, Jacquemin (fils), Meirceo, Parent, Petit (physician to the Hotel Dieu), Rey, and Villerme, at Paris, &c. &c. &c. Among these, some have very distinctly heard the simple pulsations, with hissing; others the double pulsation; and the greater number both orders of pulsation. Is it to be supposed that witnesses, so numerous, enlightened, and respectable, would all have allowed themselves to be deceived by illusory sensations? The reality of the new signs of pregnancy is, therefore, now ascertained, &c.

### 9. *Experiments on Incubation.*

M. GEOFFROY SAINT-HILAIRE has communicated to the Institute of France an interesting account of different methods by which *oviparous* animals may be rendered *viviparous.* These experiments completely succeeded with regard to *couleuvres acquatiques,* when placed in a dry situation, and under circumstances unfavourable for dropping their eggs. These were retained by the parent, and preserved in the genital receptacle; so that, at the end of a certain period of this uterine incubation, the young came forth from the eggs and the body of their mother. These experiments were not followed with equally successful results in those animals whose eggs have calcareous shells. Notwithstanding his endeavours, M. Geoffroy has not succeeded in effecting the production of young chickens alive; but he is led, from his observations, to believe that this might be done by better contrived means.

### 10. *Sympathy and Sensation.*

Dr. FODERA, in a paper read before the Academy of Sciences, has endeavoured to establish a positive distinction between these two phenomena, which have been supposed to have their common origin in nervous sensibility. His opinion is, that sensations are always effected through the medium of the nervous system; whilst sympathies are more generally phenomena of vitality, and belong to all organized beings. They are effected by continuity of texture, and are altogether independent of the nerves. In this class of facts M. Foderà ranges the movements of plants and the different communications of living organs, whether in their functions or diseases.

### 11. *Rudiments of a Fœtus in the Testicle.*

Dr. FREIDLANDER relates that a child was born in December, 1817, at a village near Glogau, which at first appeared in all respects healthy and natural. In the month of May following, it was seized with difficulty in making water, and the right testicle began to enlarge. By the 19th of June, the testicle had so much increased as to descend to the knee. On the 19th of July, a ligature was applied near the ring, which came away on the 22d, and the child did well. The testicle was four inches three lines (Rhine measure) in length, two inches four lines in breadth; its weight, seven ounces; the epidydimis was entirely wanting. After careful examination of the tunica vaginalis, a hard body was perceived, which proved to be an os femur, eighteen lines long, and without periosteum. Several other bones were subsequently found, connected, by means of cellular texture and muscular fibres, in such a manner as to form the pelvis and right lower extremity of a fœtus about four months old. The left branch of the os pubis and os ischium were wanting; the ileo-femoral articulation presented a triangular appearance; the sacrum was distinct, with its surface for receiving the first bone of the spinal column; in the middle of the pelvis was a ligamentous mass, about an inch long, resembling the rudiments of the lumbar vertebræ. The tibia and fibula were naturally formed; the interosseous ligament "a little too thick."

Several cases of this kind are on record in the works of MECKEL, DUPUYTREN, &c.; and, in confirmation of the above, it may be mentioned that it was observed by C. DIETRICH, at Glogau, a doctor, counsellor, and man-midwife!

### 12. *Croton Tiglium.*

A letter from Mr. THOMSON, of the East India Company's service, dated Dinapore, 16th January, 1822, contains the following passage:—"One of the seeds (of the croton,) reduced to powder, and made into two or three pills with a little pepper, forms the most common purgative used by the natives of this part of India. It acts speedily and powerfully; but I suspect, if not conjoined with some aromatic, it will occasion much griping. We now use it pretty frequently, combined with ginger, and, since the receipt of your letter employ the expressed oil with similar results. The latter, given in a dose of one drop, almost always purges with considerable activity; but it must be taken in some mucilaginous vehicle, for it will blister the tongue and fauces when taken by itself. I have given as much as ten drops at once, which induced instantaneous vomiting, followed by purging, but no other bad consequences ensued. In doses of three or four drops, it acts quickly and powerfully, emptying the bowels of their contents, and generally produces eight, ten, or a dozen evacuations. In certain habits and complaints, I conceive, it will prove an excellent purgative, and, from its cheapness, may be introduced with advantage into hospital practice. * * * When applied externally as a liniment, it produces a profuse crop of small pustules on the part, which may render it of service in certain chronic complaints. I have not yet, however, had sufficient opportunities of ascertaining all its medicinal properties."

### 13. *Operation for cleft Palate.*

ROUX has lately succeeded in removing this deformity in a young man, who had a natural division of the velum pendulum to so great an extent as to almost entirely deprive him of the power of articulation. By means of an ingenious operation, resembling that for hare-lip, M. Roux was enabled to bring the flap together, and procure their union in a favourable manner. He has already practised the operation, with similar success, in another case.

### METEOROLOGICAL JOURNAL.

By Messrs. William Harris and Co. 50, Holborn, London.

*From August 20, 1822, to September 19, 1822.*

| | MOON. | Rain gauge | THERM. | | | BAROM. | | DE LUC'S HYG. | | WIND. | | ATMOSPHERIC VARIATION. | | |
|---|---|---|---|---|---|---|---|---|---|---|---|---|---|---|
| | | | 9 A.M. | MAX. | MIN. | 9 A.M. | 10 P.M. | 9 A.M. | 10 P.M. | 9 A.M. | 10 P.M. | 9 A.M. | 2 P.M. | 10 P.M. |
| Aug 20 | | | 66 | 75 | 62 | 30.01 | 29.95 | 60 | 61 | SE | SE | Fine | | |
| 21 | | | 67 | 80 | 68 | 29.87 | 29.83 | 50 | 47 | ESE | SE | Cloudy | Fine | |
| 22 | | .07 | 71 | 81 | 62 | 29.80 | 29.74 | 54 | 52 | SW | WNW | Cloudy | Fine | |
| 23 | | | 68 | 73 | 53 | 29.70 | 29.81 | 57 | 43 | NNW | NNW | Fine | | |
| 24 | ☾ | .10 | 59 | 68 | 58 | 29.68 | 29.53 | 50 | 51 | N | NW | Cloudy | Fair | Rain |
| 25 | | .03 | 61 | 70 | 59 | 29.63 | 29.57 | 70 | 53 | W | SW | Fair | Sho'ry | Fair |
| 26 | | | 62 | 68 | 58 | 29.54 | 29.58 | 59 | 58 | WNW | SW | Fine | Sho'ry | Fair |
| 27 | | .07 | 63 | 69 | 57 | 29.43 | 29.54 | 59 | 63 | W | W | Fair | Sho'ry | Rain |
| 28 | | | 61 | 66 | 54 | 29.52 | 29.42 | 55 | 60 | SW | S | Overcast | Rain | Rain |
| 29 | | .02 | 58 | 64 | 55 | 29.35 | 29.51 | 54 | 58 | W var. | NW | Fine | Sho'ry | Fine |
| 30 | | .03 | 59 | 67 | 50 | 29.64 | 29.71 | 60 | 66 | N W va | WNW | Fine | Sho'ry | Fine |
| 31 | | | 53 | 66 | 54 | 29.73 | 29.88 | 51 | 61 | NNW | N | Fine | | |
| Sep. 1 | ○ | | 59 | 68 | 54 | 29.97 | 29.98 | 60 | 51 | NNW | NW | Fine | | |
| 2 | | .04 | 58 | 66 | 59 | 29.94 | 29.83 | 61 | 66 | WSW | WSW | Fine | Fair | Mist |
| 3 | | | 60 | 65 | 54 | 29.81 | 29.84 | 60 | 63 | NW | WNW | Fine | | |
| 4 | | | 56 | 63 | 56 | 29.87 | 29.93 | 53 | 61 | WNW | NW | Fine | | |
| 5 | | | 59 | 67 | 62 | 29.88 | 29.71 | 51 | 62 | WNW | W | Fine | | |
| 6 | | | 64 | 70 | 63 | 29.70 | 29.78 | 74 | 65 | WNW | WNW | Showery | Fine | |
| 7 | | | 67 | 71 | 56 | 29.83 | 29.92 | 56 | 62 | W | NW | Fine | | Fair |
| 8 | ☽ | | 60 | 67 | 53 | 29.71 | 29.77 | 53 | 57 | WSW | W var. | Overcast | | Fa r |
| 9 | | | 56 | 65 | 54 | 29.79 | 29.81 | 63 | 61 | NW | NW | Fine | Fair | Fine |
| 10 | | | 59 | 69 | 52 | 29.94 | 29.98 | 56 | 60 | NW | WSW | Fine | | |
| 11 | | | 55 | 68 | 54 | 29.78 | 29.68 | 59 | 62 | W var. | WSW | Fine | | Sho'ry |
| 12 | | .02 | 58 | 68 | 50 | 29.84 | 29.86 | 54 | 58 | NW | NE | Fair | Sho'ry | Fair |
| 13 | | | 55 | 65 | 46 | 29.91 | 29.97 | 50 | 50 | ENE | E | Fine | | |
| 14 | | | 53 | 62 | 52 | 30.06 | 30.00 | 51 | 52 | SE | ESE | Fine | | |
| 15 | ● | | 61 | 62 | 52 | 29.90 | 29.90 | 52 | 60 | E | E | Cloudy | | Rain |
| 16 | | | 59 | 65 | 54 | 29.91 | 29.94 | 63 | 60 | SW | W | Cloudy | Fine | |
| 17 | | | 60 | 72 | 52 | 29.90 | 29.90 | 68 | 69 | W | SE | Sleet | Fine | |
| 18 | | | 60 | 66 | 52 | 29.93 | 30.00 | 74 | 65 | NE | E | Fine | | |
| 19 | | | 62 | 68 | 50 | 30.00 | 29.97 | 52 | 54 | ESE | E | Fine | | |

The quantity of rain that fell in the month of August, was 1 inch 60.100ths.

